# A Computational Approach towards a Gene Regulatory Network for the Developing *Nematostella vectensis* Gut

**DOI:** 10.1371/journal.pone.0103341

**Published:** 2014-07-30

**Authors:** Daniel Botman, Eric Röttinger, Mark Q. Martindale, Johann de Jong, Jaap A. Kaandorp

**Affiliations:** 1 Computational Science, University of Amsterdam, Amsterdam, The Netherlands; 2 Université Nice Sophia Antipolis, Institute for Research on Cancer and Aging, Nice (IRCAN), UMR 7284, Nice, France; 3 Centre National de la Recherche Scientifique (CNRS), Institute for Research on Cancer and Aging, Nice (IRCAN), UMR 7284, Nice, France; 4 Institut National de la Santé et de la Recherche Médicale (INSERM), Institute for Research on Cancer and Aging, Nice (IRCAN), U1081, Nice, France; 5 Whitney Lab for Marine Bioscience, University of Florida, St. Augustine, Florida, United States of America; 6 Computational Cancer Biology Group, Division of Molecular Carcinogenesis, The Netherlands Cancer Institute, Amsterdam, the Netherlands; Sars International Centre for Marine Molecular Biology, Norway

## Abstract

**Background:**

The starlet sea anemone *Nematostella vectensis* is a diploblastic cnidarian that expresses a set of conserved genes for gut formation during its early development. During the last decade, the spatial distribution of many of these genes has been visualized with RNA hybridization or protein immunolocalization techniques. However, due to *N. vectensis*' curved and changing morphology, quantification of these spatial data is problematic. A method is developed for two-dimensional gene expression quantification, which enables a numerical analysis and dynamic modeling of these spatial patterns.

**Methods/Result:**

In this work, first standardized gene expression profiles are generated from publicly available *N. vectensis* embryo images that display mRNA and/or protein distributions. Then, genes expressed during gut formation are clustered based on their expression profiles, and further grouped based on temporal appearance of their gene products in embryonic development. Representative expression profiles are manually selected from these clusters, and used as input for a simulation-based optimization scheme. This scheme iteratively fits simulated profiles to the selected profiles, leading to an optimized estimation of the model parameters. Finally, a preliminary gene regulatory network is derived from the optimized model parameters.

**Outlook:**

While the focus of this study is *N. vectensis*, the approach outlined here is suitable for inferring gene regulatory networks in the embryonic development of any animal, thus allowing to comparatively study gene regulation of gut formation *in silico* across various species.

## Introduction

During animal development asymmetric signals set up during the early cleavage stages are utilized to initiate different pathways of cell type specific differentiation. Individual cells undergo a complex sequential and combinatorial pattern of differential activation/repression of gene activity that are causally required for the correct assignment of cell identity [1]. The body plan is thus formed by interactions between genes and proteins. A collection of such interactions defines a gene regulatory network (GRN).

A GRN can be described using mathematical models. The goal of modeling GRNs is to understand the basic properties of these networks. Various mathematical frameworks have been proposed for the description of GRNs [2]. Some models are quantitative, some models include time or spatial compartments, but combined quantitative spatio-temporal models are rare. Dynamic models that simulate quantitative gene expression levels in interacting domains can capture the formation of gene expression patterns during early animal development [3]. These dynamic simulation models are validated by their ability to reproduce spatio-temporal patterns based on experimental measurements.

**Figure 1:**
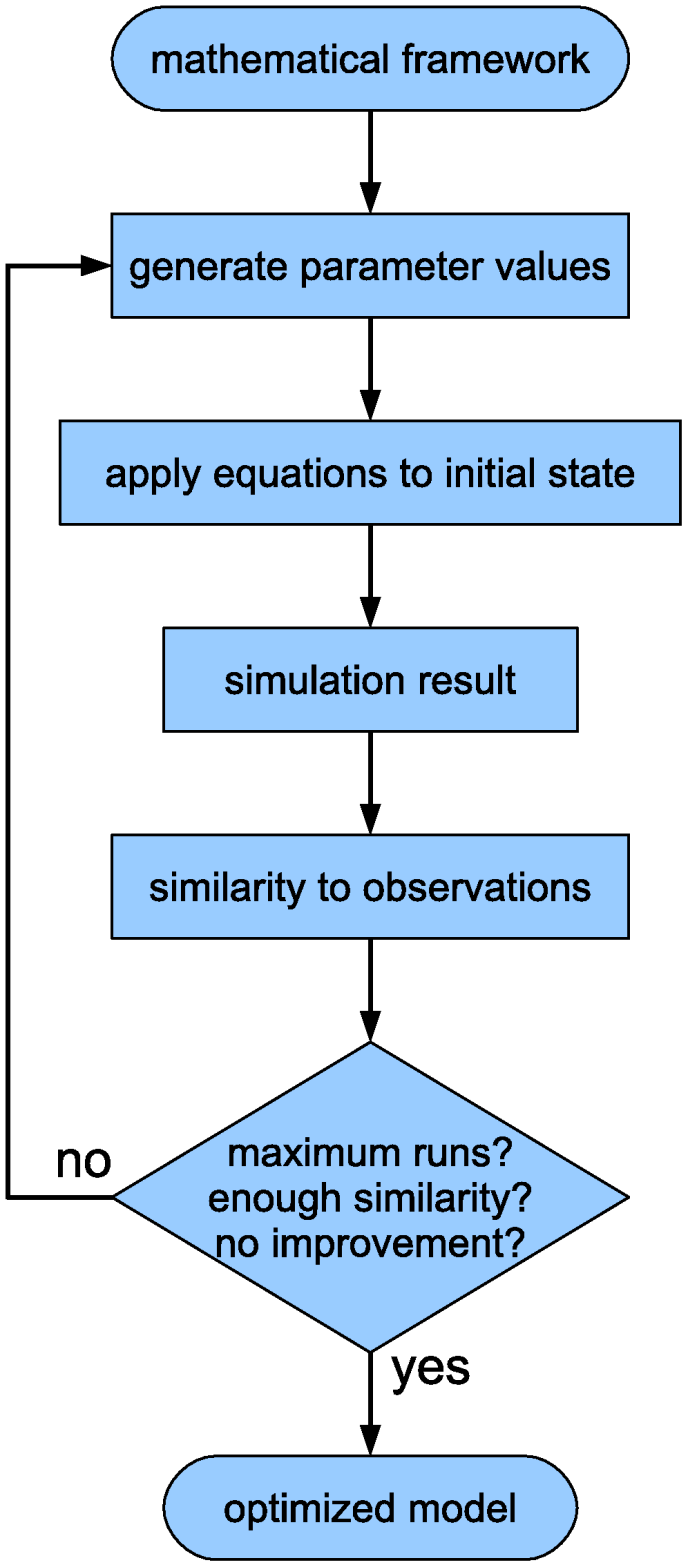
Overview of the modeling cycle. The modeling cycle starts with a framework of general mathematical equations. Initial parameter values are randomly generated or manually provided. These values are substituted into the general framework to define a specific set of equations. The equations are applied to the initial state of the system (usually derived from measurements) and produce intermediate and final states. These simulated states are compared to reference data and their similarity is determined. New parameter values are generated and new simulation runs are performed repeatedly, while stopping conditions are tested after each run (such as a maximum number of runs, a target similarity or a lack of improved similarity after multiple runs). As soon as a stopping condition applies, the cycle is terminated and the set of parameter values that results in the closest match with the observations is the optimized model. The steps that require quantitative data are encircled.

The general model building process contains three main steps [4]. First, quantitative gene expression data is required, which is extracted from spatio-temporal measurements. Second, a modeling framework is established from a set of mathematical equations. Third, the parameters in the modeling framework are estimated: the optimal parameters produce simulated expression patterns that correspond to the quantitative gene expression data. An overview of the modeling cycle is shown in Figure 1.

Modeling GRNs has the advantage that parameters can be investigated without the noise and limited precision of experimental measurements. The influence of the proposed mechanisms can be tested without the interference of many other processes that occur in living systems. Moreover, new hypotheses can be generated from abstract model properties that cannot be obtained from experimental measurements. For example, Manu et al. [5] suggested that anterior and posterior regions in the early fly embryo move towards separate basins of attraction, based on a phase space analysis of their quantitative spatial dynamic model. Biologists may regard the inferred parameters as new hypotheses for conducting further experiments. On a lower level, the quantitative extraction of spatio-temporal gene expression patterns provides a convenient method to systematically organize, analyze and share these data among workers around the world.

When modeling methods are applied to investigate GRNs, some pitfalls should be avoided. The data quality, scope and usefulness of a model should be considered.

The reliability of a numerical model depends on the quality of the data that is supplied as input. For example, RNA *in situ* hybridizations can come from various laboratories, implying that the images could be produced with different light settings, operators and purposes. Moreover, differences may also arise from variation among individual samples.

Conclusions beyond the scope of the model should also be viewed with caution. If a model identifies spatial and temporal correlations between pairs of genes, then these should not be treated as interactions, even though direct or indirect influences would be the most straightforward cause of these correlations. However, these proposed interactions can be directly tested experimentally thus vetting the model's predictions.

Finding an optimal solution in problems with many unknown parameters can be computationally extremely intensive. Besides, the optimal solution is not necessarily the best approximation of the biological system. Analyzing multiple solutions from a repeated stochastic search to determine which parameters are most consistent (and therefore most reliable) is an alternative method. An analysis of many solutions can provide more information than the best solution from a single optimization run.

Currently, the most precisely described spatio-temporal regulation mechanism for early development is the gap gene network in the fruit fly *Drosophila melanogaster* [6], [7]. One notable insight is the function of cross-regulatory interactions among gap genes [8]. These interactions are necessary for precise gap gene expression domains to emerge from a larger spread in maternal concentration gradients.

In comparison to most other metazoans, gene regulation in early fly embryos such as *Drosophila melanogaster* is easy to understand, because the regulatory proteins do not require intermediate metabolites to interact with the DNA [9]. These straightforward regulatory interactions are coupled to the early fly morphology: no membranes are present during the first nuclear division cycles, so transcription factors can diffuse between nuclei. In other metazoan embryos, complex signaling pathways operate from the early cleavage stage cells, and many regulatory interactions are mediated by a chain of inter- and intracellular compounds.

Even after the formation of membranes around the nuclei, the fly embryo outline does not change much due to its encapsulation in the eggshell. This allows a highly automated procedure for image segmentation and expression profile extraction [10]. However, the shape of most other metazoan embryos changes continuously, especially during blastula formation and gastrulation.

**Figure 2:**
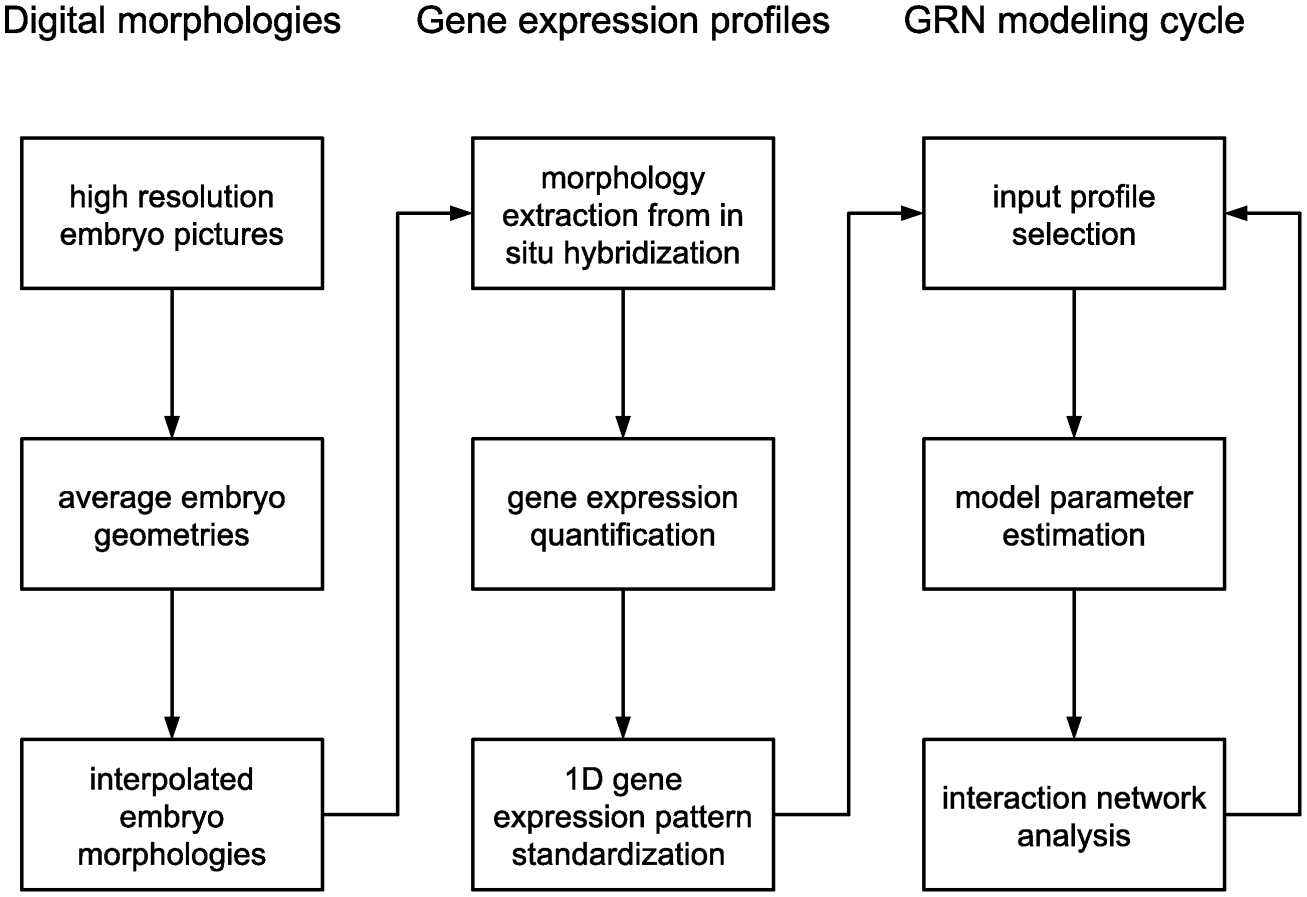
Overview of the GRN production pipeline. The pipeline is divided in three main parts, which are required for the study of pattern formation in any system, and nine smaller steps that apply specifically to complex and changing shapes. The main parts are the design of digital morphologies, the preparation of standardized gene expression profiles and the implementation of gene regulation models. For complex and changing morphologies, the particular steps are explicitly mentioned. First, embryo micrographs are prepared with a sufficiently high resolution to observe the tissue outlines. The outlines in every time bin are averaged to obtain representative embryo geometries for all developmental stages of interest. Points for an approximate spline of each geometry are selected and these spline points are interpolated for subsequent geometries to obtain a continuous series of digital embryo morphologies. The second part is the preparation of expression profiles from observed gene expression patterns, starting with the adjustment of a digital morphology to a gene expression image such as an in situ hybridization. The spatial gene expression is quantified by measuring the expression signal along the adjusted morphology. The raw signal is edited and interpolated at a fixed number of equidistant points to arrive at a standardized gene expression profile. In the third part, the gene regulatory network is inferred and updated. A set of expression profiles is selected as modeling input. The free parameters of a network model are estimated with an optimization algorithm. The optimized parameters are incorporated in an interaction network that can be analyzed and validated; the modeling cycle is then repeated with new conditions.

An extended pipeline is proposed with the purpose of elucidating gene regulation mechanisms in other animals beyond flies. The particular steps in this pipeline, summarized in Figure 2, already provide means to quantitatively compare external properties like average shapes and expression patterns among different species. The complete procedure may eventually allow the comparison of pattern formation programs.

**Figure 3:**
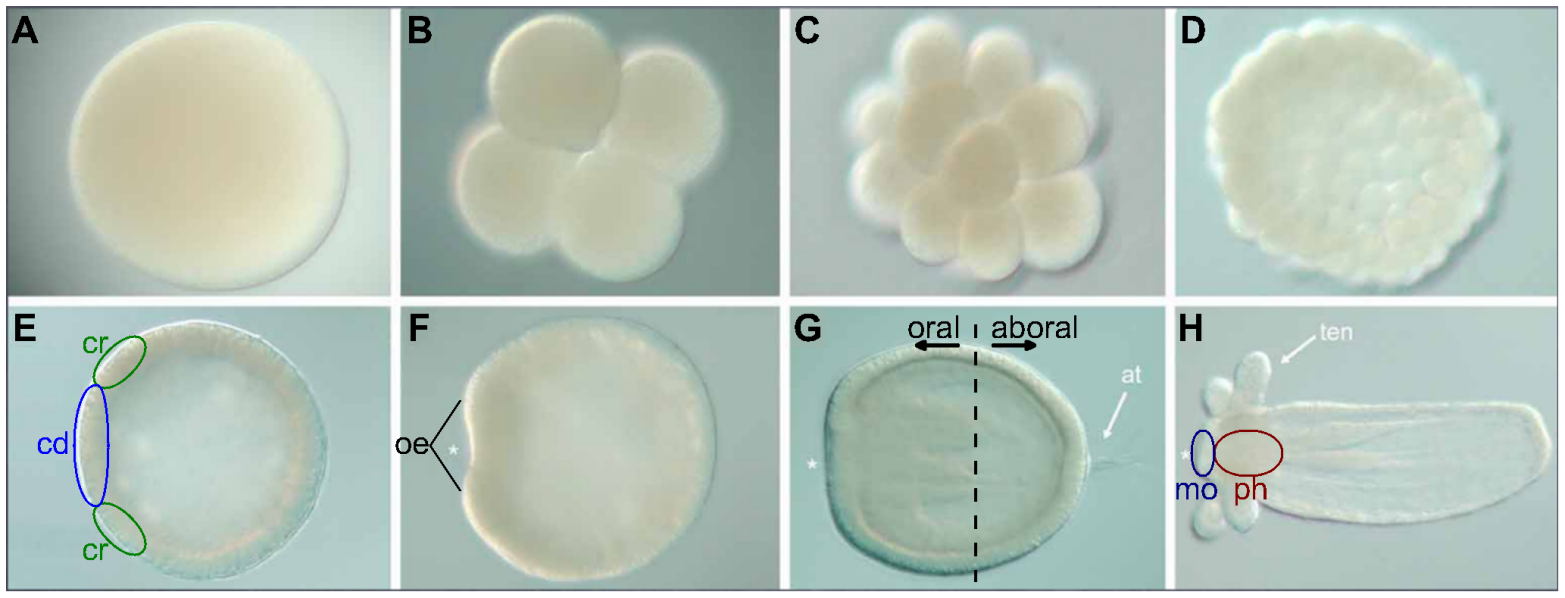
Various stages of *N. vectensis* embryonic development. Development stages from egg to polyp are shown, with the oral pole to the left in panels F-H (indicated with an asterisk). A) Fertilized egg (0 h). B) Four-cell stage (3 h), often after two cleavages finish simultaneously. C) 32-cell stage (5 h). D,E) Cleavages result in a hollow sphere called a blastula (10–20 h). F) Invaginating cells at the oral pole mark the beginning of gastrulation (24 h). G) Planula larva (72 h) with a double cell layer and apical tuft (at). Black arrows indicate the oral and aboral directions. H) Juvenile polyp with four early tentacles (ten). (cd  =  central domain, cr  =  central ring, mo  =  mouth, oe  =  oral end, ph  =  pharynx. Development times in hours at 16°C estimated from [20], [30].)

In the current study, the starlet sea anemone *Nematostella vectensis* (hereafter referred to as *N. vectensis*) is used as a case study to investigate GRNs during embryonic development. As a model organism, *N. vectensis* is very convenient since it is sufficiently small and transparent for use with various microscopy methods, it is easily grown in a petri dish and it can reproduce sexually and asexually in a laboratory environment [11]. Also in terms of development, *N. vectensis* is an interesting model organism, as its mode of gastrulation is common among metazoans and many conserved signaling pathways have been identified, while its body plan is relatively simple (Figure 3H) [12].

Many gene expression images have been published for *N. vectensis*, and some papers are listed at the Cnidarian Evolutionary Genomics Database [13]. An increasing number of raw pictures, including unpublished material, is collected in the marine invertebrates database Kahi Kai [14]. While these images show the spatio-temporal progress of gene expression patterns, they do not give insight into how these complex patterns arise.

Previously, a method was described for the quantitative extraction of gene expression patterns in embryos with a changing morphology [15], such as *N. vectensis*. The availability of such a method is a basic requirement for modeling spatio-temporal gene regulation and forms the first step towards GRNs for morphological development in various animals. Still, an understanding of the dynamical aspects of these GRNs requires a more precise description of the complex signaling between genes and among cells.

Therefore, in this study we first apply the above method on microscopy images of RNA hybridizations and protein antibodies, and quantitative PCR (qPCR) measurements, such as obtained from the Kahi Kai database. Having quantified these images, a high-level mathematical description is sought to understand what properties and interactions are required for gene products to exhibit these spatial distributions during the embryo's progressing development. We start with a minimal set of genes to explain the appearance of characteristic features in the quantified expression patterns. We assume that initially studying a small number of genes will provide a clear view on the major mechanisms, while refining a model by adding more genes should show additional mechanisms responsible for properties like stability and fixed final expression domains.

We focus on *N. vectensis* gut formation. The gut is formed from an embryonic tissue called endoderm, and the delineation of endoderm (internal tissue) from overlying ectoderm (giving rise to the outer epidermis of the animal) is among the first visible developmental events in sea anemone development. To select a set of genes for simulation, we first determine which of the genes that are involved in the process of endoderm formation display similar behavior by clustering genes with similar patterns. We assume that selecting a single member from each cluster of genes is usually sufficient to discover the main mechanism that can then be elaborated on with additional genetic information. When a main mechanism has been elucidated, additional genes can be selected by narrowing the cluster sizes.

**Figure 4:**
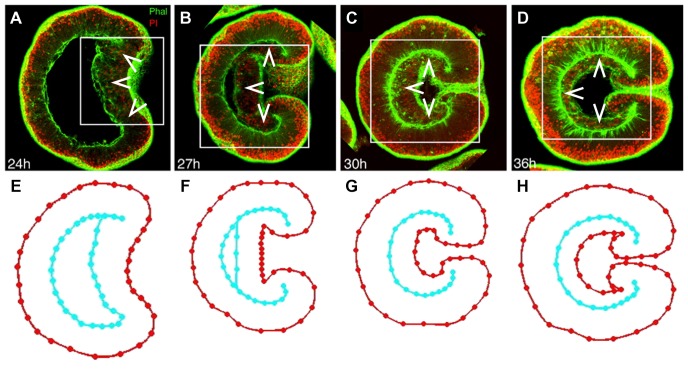
Graphical embryo morphologies derived from confocal microscopy images. A-D) *N. vectensis* embryos were stained at various stages of gastrulation with fluorescent markers for filamentous actin (phalloidin in green) and nuclei (propidium iodide in red). Arrowheads indicate the endoderm. The embryos are oriented with the blastopore to the right. Development times after fertilization at 16°C are indicated in the lower left corner. The images are modified from [20]. A) Cells at the oral pole are invaginating. B, C) The inner cell layer (endoderm) is zipping up with the outer layer (ectoderm). D) The endoderm is flattening against the ectoderm. E-H) Based on these confocal cross sections, average cell layer geometries have been constructed. The cell layer outlines are closed loops; the inner loop overlaps itself where endoderm and ectoderm are zipped up. Only a selection of confocal micrographs and embryo geometries is shown. More details on the construction of these geometries are given in Methods.

**Figure 5:**
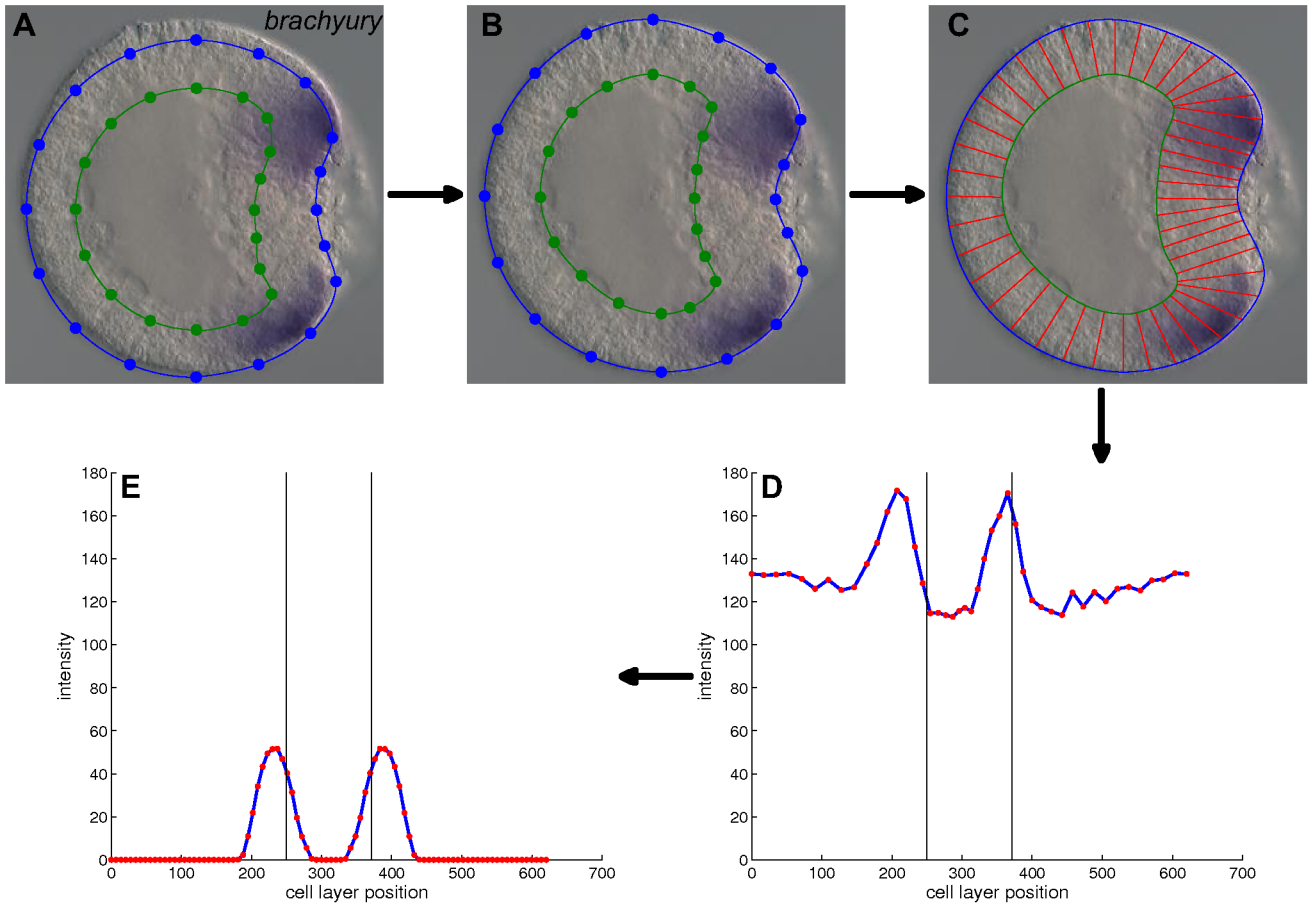
Gene expression quantification procedure. A) An *in situ* hybridization of the gene *brachyury* (blue reaction product) of an *N. vectensis* embryo at the correct developmental stage oriented with the site of gastrulation to the right (modified from www.kahikai.org). The overlaid prototype morphology has not been adapted. B) The morphology has been adjusted to the observed cell layer outlines. C) The cell layer is decomposed into segments of approximately the user-defined width. D) For each segment the average color intensity is calculated (the red intensity profile has been selected in this example). Expression intensity is measured along the cell layer and plotted against the cell layer position. E) Gene expression profile after editing the raw intensity profile. The vertical black lines correspond to the position of the oral pole.

Our general approach consists of three basic steps: 1) design of digital morphologies, 2) quantification of spatial expression data and 3) mathematical analysis. For the model organism *N. vectensis*, a range of morphologies are derived from high-resolution confocal microscopy pictures during the first three days of embryonic development (Figure 4). These morphologies are then applied as adaptive masks to quantify expression intensity from RNA *in situ* hybridization and protein immunolocalization images of *N. vectensis* development (Figure 5). To infer the regulatory gene network, selected expression profiles from four genes at three distinct time points serve as reference data in the gene circuit model. Genetic interactions that show the same sign in many optimization runs are incorporated in a regulation network.

The primary objective of this paper is setting up a flexible and complete workflow to obtain putative regulatory information from gene expression images at multiple time points. Current limitations are stated with propositions for improvements. In this preliminary study, we arrive at a rough network structure of regulatory interactions in the gut formation of *N. vectensis* during early development. This regulation network will be improved based on new expression data that have become available recently.

## Results

A set of conserved genes that are expressed in cnidarians and echinoids is provided in [16] (Figure 4 herein) and these genes are ordered in functional modules that are associated with tissue differentiation. A single functional module is an interesting starting point for studying gene regulation, because a functional module may act as a regulatory module as well. The genes that are associated with gut development are useful for two-dimensional quantification, because their expression patterns are cylindrically symmetric. Based on the list of conserved genes for gut development, a total of 70 gene expression micrographs for 13 genes from *N. vectensis* have been retrieved from various sources. No gene expression images have been found for *six1/2* and *blimp*, while *gata* displays a grainy pattern during gastrulation [17], which is unsuitable for quantification.

**Figure 6:**
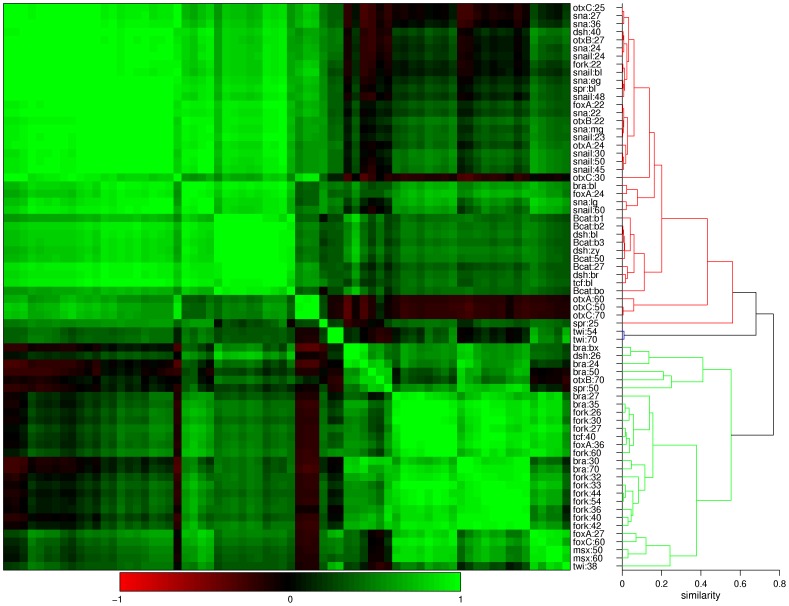
Correlation matrix and dendrogram of expression profiles for gut development genes. The 70 profiles listed in Dataset S1 are clustered using Pearson correlation and unweighted average linkage. The color scale goes from red (negative correlation) to black (no correlation) to green (positive correlation). The dendrogram is cut off at linkage distance 0.6 to obtain the three clusters colored in green, red and blue. (Here, the linkage distance is one minus the correlation coefficient.)

**Figure 7:**
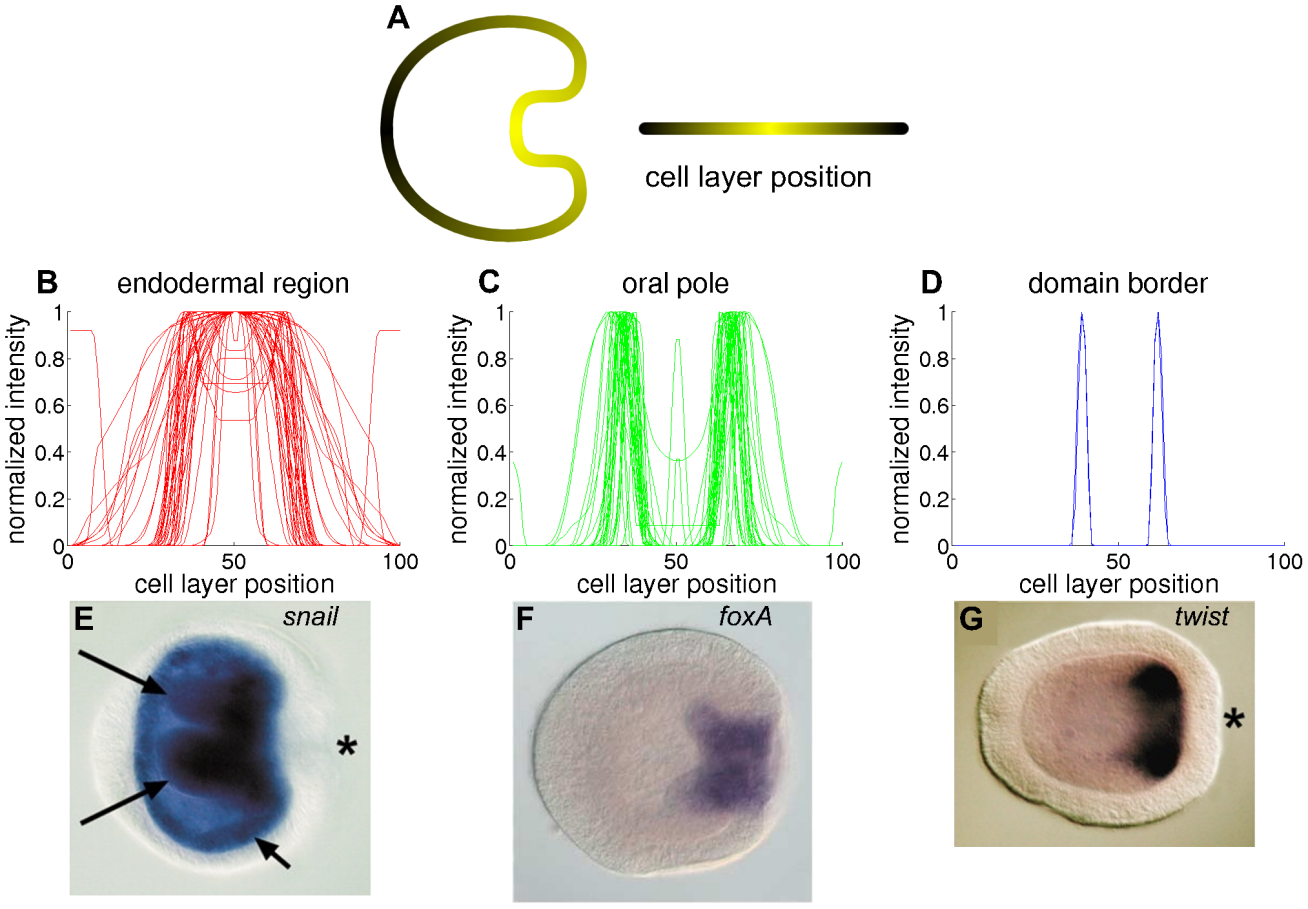
Spatial gene expression profiles divided in three hierarchical clusters. A) The embryo cell layer is mapped to the horizontal axis. B-D) The spatial expression profiles plotted in each graph show common features within the clusters from the dendrogram in Figure 6. B) Genes in the red cluster are mainly expressed in the endodermal region (segments 40 to 60). C) Genes in the green cluster show expression at the oral pole (segments 30–40 and 60–70). D) The remaining gene is expressed in a narrow domain that roughly corresponds to the border of both regions (right). These clusters are applied for the selection of genes that are used in the simulations. E-G) *In situ* RNA hybridizations from each cluster in the planula stage: *snail* (E), *foxA* (F) and *twist* (G). The annotations appear in the original publications [17], [31]; the meaning of these annotations is irrelevant for the quantification procedure.

The 70 gene expression images and the derived spatial expression profiles for the cluster analysis are listed in Dataset S1. The correlation matrix and dendrogram are displayed in Figure 6. Applying a similarity cut-off of 0.6 (one minus the correlation coefficient), the genes are clustered in three groups, for which the profiles are plotted in Figure 7. The largest branch, colored in red, contains 40 profiles characterized by their expression in the endoderm. The green branch contains 28 profiles characterized by expression in the (presumptive) oral region. The two remaining profiles in the blue branch correlate with both clusters, as these consist of sharp peaks at the edges of both regions.

Expression domains for a large set of genes in the *N. vectensis* blastula have recently been analyzed with *in situ* hybridization [18]. Expression was found in a central domain at the oral pole, in rings at various distances from the oral center and in an aboral region. The genes in our cluster analysis correspond to those that are expressed either in the central domain or in the central ring (these regions are indicated in Figure 3E). Even though our analysis contains relatively few measurements from the blastula stage, the strong correlation within the two main clusters agrees with this classification. Note that some genes appear in both clusters; this could be caused by dispersion of the staining agent in older images, while some genes are repressed in the central domain after initially being expressed in this area. The separation into these two clusters also suggests that the central domain develops into endodermal tissue, while the central ring becomes the future mouth and pharynx (Figure 3H). Cell fate experiments are however required to confirm this observation.

The expression profiles within each cluster are strongly correlated (Figure 6) and show substantial overlap (Figure 7). Half of the genes have expression profiles in multiple clusters. In the blastula and early gastrula stages, the separate domains have partially overlapping lines of sight. Moreover, oral views reveal that these expression domains can have irregular shapes [18], causing a variable domain boundary among individuals.

In Table 1, the primary spatial expression feature for each gene is indicated at the cleavage, gastrula and planula stages. Because many genes appear in two clusters in the gastrula stage, the expression cluster for each gene is only indicated for the planula stage. Based on the stage they first appear and on their spatial expression in the planula, four groups of genes are identified. First, β-*catenin*, *dishevelled* and *tcf* are expressed already at the early cleavage stage. Second, *otxA*, *otxC* and *snail* are present in the gastrula and expressed in the planula endoderm. Third, *brachyury*, *foxA*, *otxB* and *sprouty* are expressed at the oral pole in the planula. Fourth, *foxC*, *msx* and *twist* are not yet expressed at the gastrula stage. To arrive at a set of profiles to be used for simulation, we selected from each cluster the single gene with the largest number of expression profiles, namely β-*catenin*, *snail*, *foxA* and *twist*.

**Table 1:** Expression pattern properties for conserved gut development genes in *N. vectensis*.

		genes												
		group 1			group 2			group 3				group 4		
**stage**	**expression**	Bcat	dsh	tcf	otxA	otxC	sna	bra	foxA	otxB	spr	foxC	msx	twi
cleavage	present^a^	X	X	X										
gastrula	present^a^	X	X	X	X	X	X	X	X	X	X			
planula	endoderm^a^	X	X		X	X	X							
	oral pole^a^			X				X	X	X	X	X	X	

a In the cleavage, gastrula and planula stages, the presence and location of the main expression domain is indicated for each gene. The “endoderm” and “oral pole” designations are based on the hierarchical clusters (see text).

For β-*catenin*, a profile is selected as a maternal gradient, because its expression precedes the gene expression in the other groups. For the purposes of the model, this means that β-*catenin* is initiated with a nonzero profile that remains constant. The other genes are initiated with an expression level of zero.

**Figure 8:**
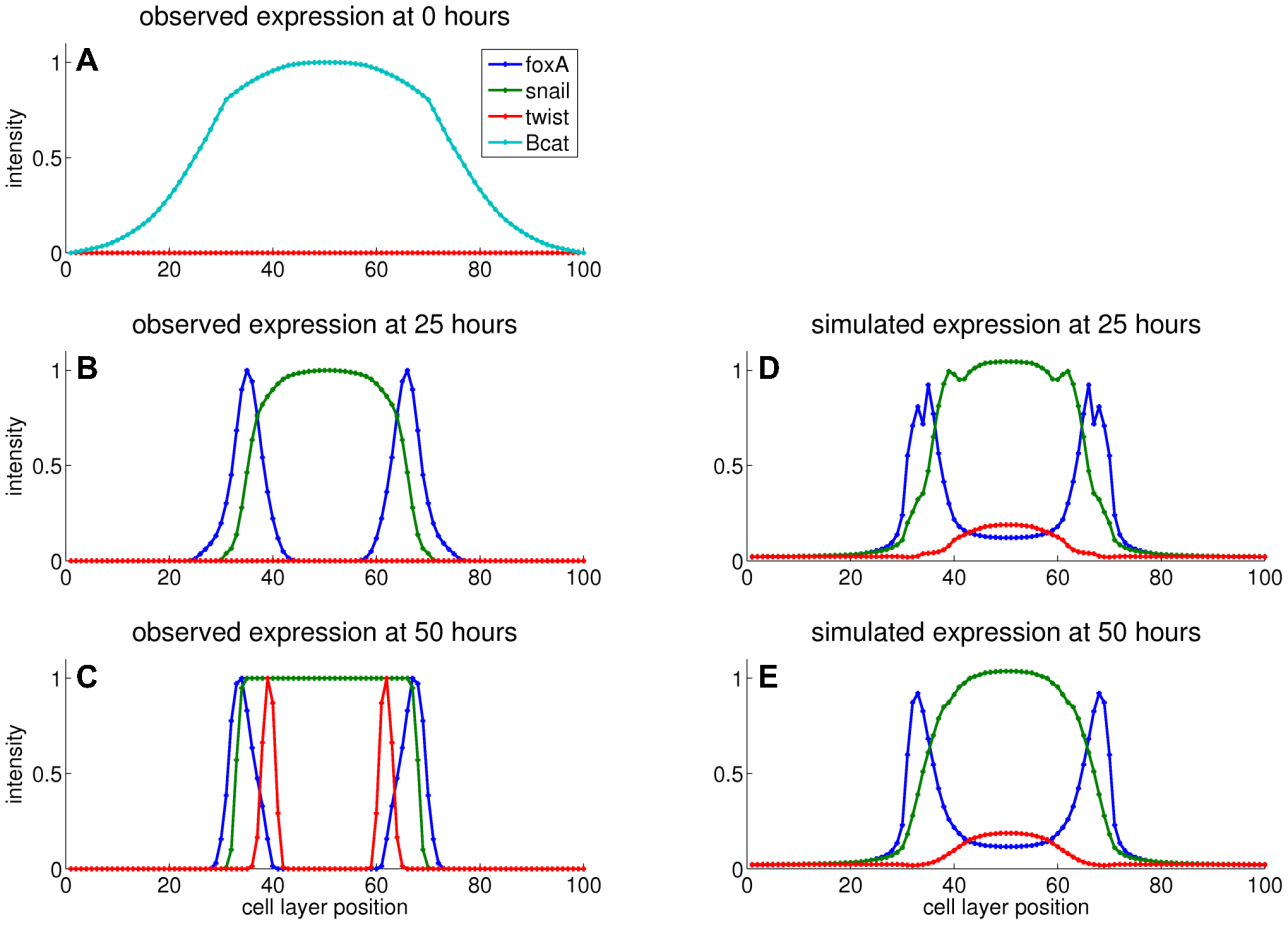
Simulated profiles compared to observations. A) All simulations start with the interacting genes *foxA*, *snail* and *twist* unexpressed and a gradient of β-*catenin*. B) At 25 hours, *foxA* and *snail* are expressed in domains near the center. C) At 50 hours, sharply bound twist expression occurs within the *snail* domain. The bounds of *foxA* and *snail* expression are sharper as well. D, E) The best simulation model approximates the positions of the *foxA* and *snail* domains, while the late twist peaks are not reproduced. The β-*catenin* gradient is kept unchanged in both the reference and the simulations, therefore this profile is not displayed in the middle and bottom plots.

**Figure 9:**
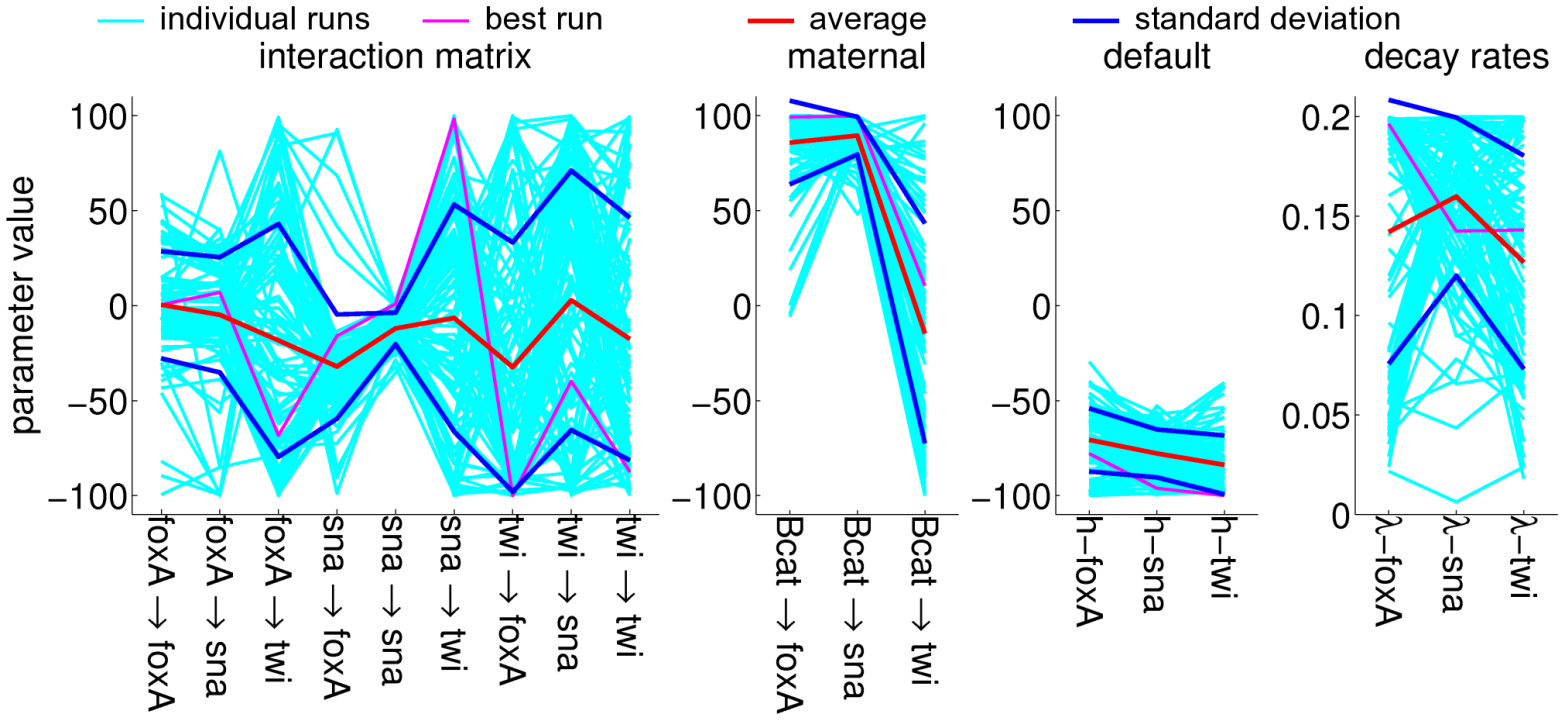
Parameter sets from 100 optimization runs. From left to right, the elements in interaction matrix *T*, the maternal influences *m*, the default influences *h* and the decay coefficients λ. The best fit is displayed in Figure 8.

**Figure 10:**
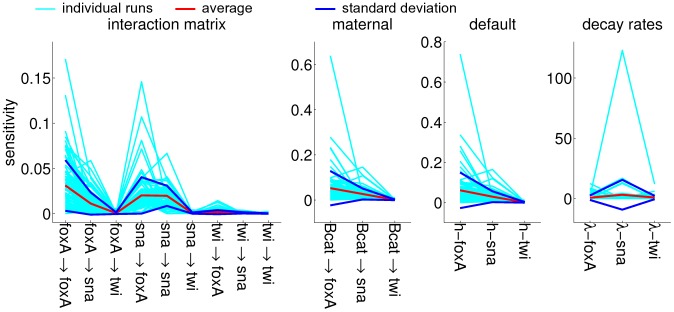
Parameter sensitivities for the parameter sets in Figure 9. The sensitivity is calculated as the highest average derivative of all concentration profiles to the parameter value (see Methods). From left to right, sensitivities of the elements in interaction matrix *T*, of the maternal influences *m*, of the default influences *h* and of the decay coefficients λ.

**Figure 11:**
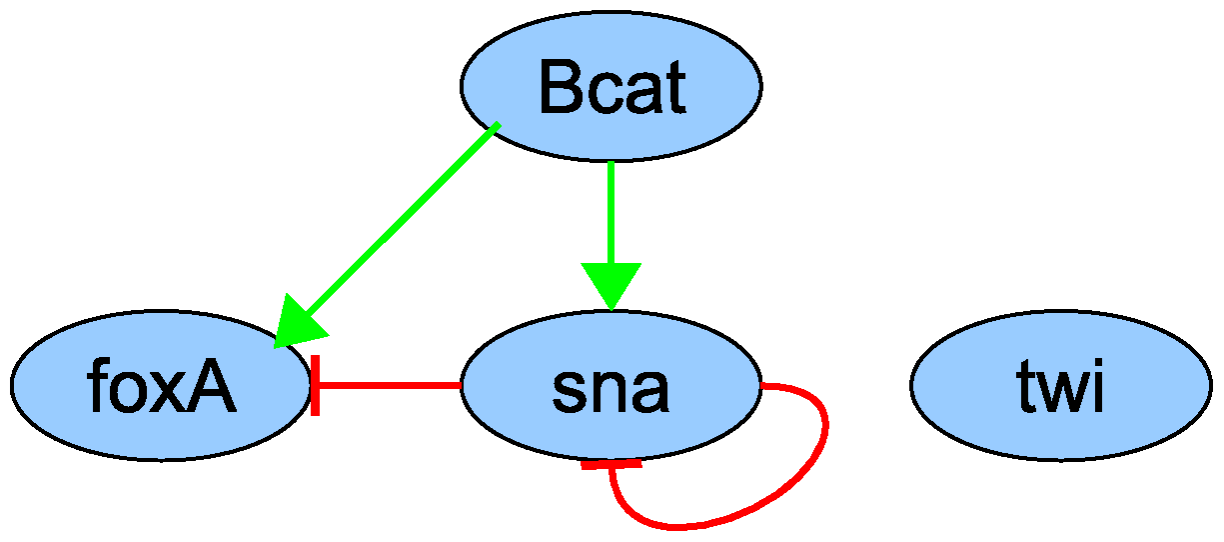
Proposed gene regulation network for gut development in *Nematostella vectensis*. The connections in this network are based on the estimated parameters in a simplified gene circuit model from 100 parameter estimations from 0 to 50% of the estimated sets are incorporated into the network. The genes in this network represent groups of spatially correlated genes, as indicated in Table 1.

The input profiles for parameter estimation are listed in Table 2, with all non-constant profiles initialized at zero and a constant maternal profile. These profiles are displayed in Figure 8 and compared to the simulated profiles from the model with the highest similarity. The parameter sets from every run (100 runs in total) are collected in Figure 9 and the parameter sensitivities are plotted in Figure 10. If an interaction parameter is positive in at least 90% of the estimated parameter sets, a corresponding activation is indicated in the regulation network (Figure 11). Likewise, an inhibition is added for an interaction parameter that is negative in 90% of the sets or more.

**Table 2:** Gene expression profiles selected for simulations.

Interacting genes^a^	Maternal gene^a^	Timepoints^b^		
		0 hours	25 hours	50 hours
*foxA*		zeros	fork:26	fork:44
*snail*		zeros	snail:24	snail:45
*twist*		zeros	zeros	twi:54
	β-*catenin*	Bcat:b2	Bcat:b2	Bcat:b2

a The interacting genes are included in the interaction matrix, while the maternal gene is merely a regulator with a constant profile. The four genes are selected from the groups indicated in Table 1 (see text).

b The expression profiles at 0 hours serve as initialization for the simulations. The profiles at 25 hours and 50 hours are used as fitting targets to rank the simulation models. The table entries refer to the profiles in Dataset S1 and are plotted in Figure 8. “zeros” means no observed expression and represents a list of zeros.

Figure 8 shows that the simulated *twist* expression pattern deviates most from the observed expression pattern: both simulated *foxA* and simulated *snail* patterns display peaks at the observed locations, while a shallow simulated *twist* band appears at the incorrect location. *twist* expression is observed only at the last time point and even then the area under the peaks is smallest for *twist*, so correctly simulated *twist* peaks would contribute the least to the overall similarity. Moreover, the *twist* peaks are located within the central region of both the β-*catenin* and *snail* domains, so no agent is present to induce a separation in the *twist* domain. Compare this to the simulated *foxA* peaks that are induced by activation from β-*catenin* and repression from *snail* (purple line in Figure 9).

Because the simulated *twist* pattern shows the worst fit with the observed pattern and maintains the lowest expression levels over the whole length, it is expected that the parameters that involve the twist gene are the least sensitive. The graphs in Figure 10 show that this is indeed the case.

**Figure 12:**
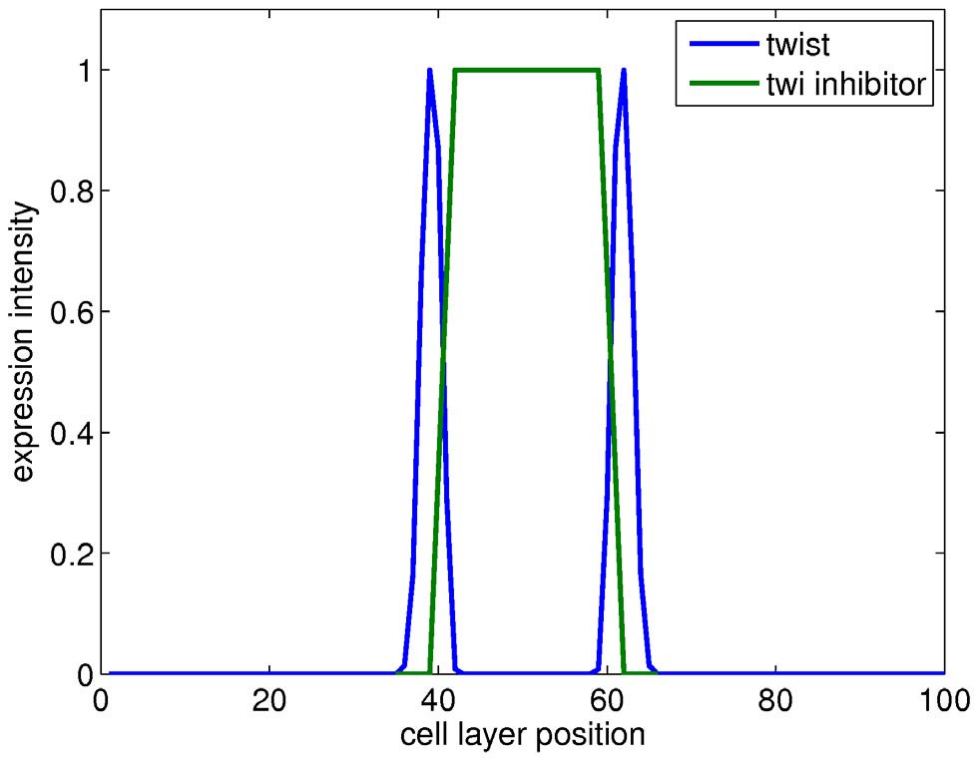
Approximated pattern for *twist* inhibitor. A gene that is expressed in the aboral endoderm is necessary to suppress the *twist* gene and limit *twist* expression to the oral endoderm.

In the simulation, *twist* is upregulated early (at 25 hours), and at the wrong location (in the aboral endoderm). A gene that is expressed in the aboral endoderm is needed to limit a *twist* peak to the oral endoderm (Figure 12). This role might be fulfilled by *otxA*, *otxB* or *otxC*, but another gene that is not necessarily conserved could serve this purpose as well.

Our results are not conclusive, so a comparison with a gene network from another organism would not yield new knowledge. Still, the model may allow initial comparisons with observations in sea urchins.

For example, the regulation of endomesoderm formation in the sea urchin is intensely studied [19]. The extensive network shares the genes β-*catenin*, *brachyury*, *foxA*, *otx* and *tcf* with our limited study. The reported interactions in the sea urchin system are listed in Table 3, along with a comparison to the inferred edges in the sea anemone regulation network. From this comparison, it seems that the regulatory function of otx in sea urchins is more similar to otxB than to otxA or otxC in sea anemones. The correspondence of most relations in sea urchin and sea anemone is remarkable, although no strong conclusions can be drawn.

**Table 3:** Experimental influences in the sea urchin [19] compared to inferred interactions in the sea anemone.

Sea urchin	Sea anemone	Agreement?
bra activates *foxA*	*brachyury* and *foxA* are clustered	yes
bra activates *otxB*	*brachyury* and *otxB* are clustered	yes
foxA represses *foxA*	*foxA* cluster lacks interaction with itself	no
otx activates *bra*	*brachyury* and *otxB* are clustered	ambiguous
	*otxA/C* cluster likely represses *brachyury* cluster	
otx activates *foxA*	*foxA* and *otxB* are clustered	ambiguous
	*otxA/C* cluster represses *foxA* cluster	
otx activates *otx*	*otxB* cluster lacks interaction with itself	no
	*otxA/C* cluster represses itself	
tcf/β-catenin activates *bra*	β-*catenin* cluster activates *brachyury* cluster	yes
tcf/β-catenin activates *foxA*	β-*catenin* cluster activates *foxA* cluster	yes

## Methods

### Digital morphologies from high-resolution micrographs

*N. vectensis* embryos at 16°C were stained with propidium iodide and phalloidin to visualize nuclei and cell boundaries, respectively, and imaged with a confocal microscope [20] (Figure 4A-D). These images were used to generate nodes placed along the cell layer boundaries to indicate their shapes over developmental time points. Multiple samples (2 to 5) were recorded and node locations are averaged to generate representative geometries for all time points (Figure 4E-H). This averaging reduces the influence of local irregularities in individual embryos. Strategic points are picked from these average geometries for interpolation of subsequent geometries, to obtain a continuous range of embryo morphologies.

### Standardized profiles from gene expression images

Published and raw gene expression images are imported into GenExp, a Matlab interface to quantify gene expression patterns. The expression profile is extracted as described in [15]. A prototype morphology is overlaid with the image (Figure 5A). The selected morphology is adapted to the observed embryo's cell layer by dragging the points of the digital morphology over the cell layer boundaries (Figure 5B). The cell layer is decomposed into segments with edges between the inner and outer cell layer boundaries (Figure 5C). The average color intensities of the pixels within each segment are plotted as a function of the segment's position on the cell layer (Figure 5D). This plot is edited to compensate for artifacts from the environment, annotations and decomposition (Figure 5E). The edited plot is interpolated at a hundred equidistant points and the intensity is scaled to unity to arrive at a standardized expression profile suitable for numerical analysis.

### Numerical analysis

#### Spatial correlation and gene selection

All standardized expression profiles are clustered with average linkage and Pearson correlation distance; these measures are straightforward and applied most frequently in co-expression analysis [21]. In a table, the genes are ordered based on the characteristics of their expression profiles at the cleavage, gastrula and planula stages. From each group of genes with similar expression characteristics, a gene is selected for simulation. Expression profiles of the selected genes at roughly 0, 25 and 50 hours after fertilization serve as input for the parameter estimation.

Parameter estimation. The gene circuit model [4] (derived from the connectionist model [22]) is a mathematical framework that can simulate gene regulation in flies with no prior knowledge about interaction mechanisms. It is based on the assumption that the gene products influence the production rate of proteins, while diffusion and decay are protein-specific. The general differential equations for the protein concentrations in a one-dimensional chain of nuclei are
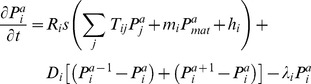
(eq. 1)with product concentrations *P* of gene *i* in nucleus *a*, interaction matrix *T*, maternal influence *m* of maternal gene *mat*, constant influence *h*, sigmoid function *s*, production rate *R*, diffusion coefficient *D* and decay rate λ. For each gene *i*, the value of parameters *T_ij_*, *m_i_*, *h_i_*, *R_i_*, *D_i_* and λ*_i_* need to be determined.

The modeling cycle is summarized in Figure 1. Parameter values are obtained by simulating a system with many sets of trial parameters from an initial state and comparing the simulated concentration profiles to the observed reference data.

We simplified the gene circuit formalism for the simulation model:
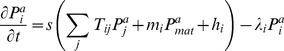
(eq. 2)with parameters *T*, *m*, *h* and λ, and sigmoid function *s*(f)  =  ½ + arctan(f)/π. The main motivation for removing the diffusion and replacing the production rate with a constant is our focus on the interaction parameters *T* and *m*. Moreover, our one-dimensional expression profiles are simplifications of two-dimensional curved surfaces. The relative amount of cells that is mapped to each point depends on the embryo's spacial shape, and this shape is changing during the embryo's development. Therefore, the diffusion function would be time dependent and too complicated for our simple model. Neglecting the diffusion is justified based on the insensitivity of the diffusion coefficients in *Drosophila* gene circuits [23]. The production rate would correlate strongly with the decay rate, and this would result in a superfluous expansion of the search space. The production rates in *Drosophila* gene circuits are insensitive as well.

With this simulation model, a hundred optimization runs are performed, minimizing the root-mean-squared value of differences with the reference profiles at 25 and 50 hours. We applied the enhanced scatter search (eSS) algorithm [24] with at least 10,000 function evaluations for each run and the local search options deactivated. The eSS algorithm performed well for high-dimensional benchmark problems in comparison to other methods [25].

**Regulation network inference.** The best parameter set found in each optimization run is collected for a statistical analysis. The values from these parameter sets are displayed in a scatter plot and standard deviations around the means are indicated.

The parameter sensitivities from the optimized sets are calculated with the iterative approximation based on directional derivatives [26]. A parameter's sensitivity is the change in the system with a changing parameter value. The derivative of a protein concentration to the parameter value is a measure for the parameter sensitivity. The algorithm calculates the derivatives of all concentrations with respect to the parameter value for every point along the cell layer. The derivatives are averaged along the cell layer for each concentration and the highest average derivative is defined as the system's sensitivity towards this parameter. The sensitivities are plotted for all parameters in the optimized sets, along with the mean sensitivity values and standard deviations (Figure 10).

Those interaction parameters that have an equal sign for 90% of all values are incorporated into the proposed regulation network. These parameters are expected to be most significant, so they should exhibit high sensitivities.

## Discussion

### Gene expression quantification issues

Embryonic tissue is expanding during development, but this expansion is not homogeneous. Static points on the embryo geometries are mapped to fixed positions to minimize the apparent shift of expression patterns due to different growth rates in the embryo body. The fixed points are located roughly at the oral end after gastrulation has commenced (this region is indicated in Figure 3F), because many genes display a stable expression domain around this point and this location is readily established. Without a correction for inhomogeneous tissue expansion, expression at the oral ectoderm would be displaced toward the aboral ectoderm in the one-dimensional cell layer during gastrulation. The uncorrected patterns would exhibit less correlation over time and model parameters would be inferred to accommodate the imaginary shift, while the expression remains at the same position in three-dimensional space.

All quantified expression intensity is normalized to unity, because the raw intensity of *in situ* RNA hybridizations depends on the duration of hybridization, which is different for separate measurements. As a consequence of the normalization, the absolute expression levels between genes and developmental stages cannot be compared. All analysis is based on the differential gene expression within the individual embryos, which means that the strength of regulatory interactions between genes cannot be determined. The sign of the interaction parameters can only be inferred if the regulatory interactions do not influence the maximum expression. A justification for this strong assumption is that the simulated genes are selected for their expression in different domains. To enable more accurate simulations, the quantified gene expression patterns can be combined with information from qPCR measurements.

If spatial information is available for proteins, then this is used rather than mRNA distributions, because proteins are the compounds with actual regulatory function. For *dishevelled*, both protein antibody stainings, fluorescent protein constructs and *in situ* RNA hybridizations in *N. vectensis* were available, and therefore only the protein pictures have been analyzed. Moreover, *dishevelled* transcripts are uniformly expressed throughout embryonic development [27], so *dishevelled in situs* do not provide information on differential regulation. For β-catenin, only protein distributions are available.

From the *in situ* measurements consulted in this study, the initial time of expression is hard to determine for each gene. The measurements are not part of a systematic time series, so an approximate time of development is derived from the embryo morphology. For the majority of genes that make their first appearance during the blastula stage, this time can only be classified as roughly as 10 to 20 hours after fertilization. Besides this lack of precise timing, the *in situ* hybridization technique is quite insensitive to low expression levels. These limitations are reduced with the availability of systematic measurements in the blastula stage and highly sensitive qPCR data. These quantitative measurements can be applied to define the total amount of mRNA in an embryo. This amount is then approximated as the total expression intensity in quantified gene expression patterns from *in situ* hybridizations.

### Proposed improvements for geometry extraction

Currently, the geometry extraction procedure is very labor intensive and time consuming. For fly embryos, algorithmic image segmentation speeds up this task substantially [10]. For *N. vectensis* embryos, an extended image segmentation method would be required to take irregular shapes and low-contrast internal structures into account. Such an extended image segmentation method could significantly reduce the manual effort to identify the cell layer boundaries. Algorithm-guided image segmentation would also reduce subjective human judgment in estimating the boundaries, especially for rough or blurred edges. Raw RNA hybridization images of *N. vectensis* and other marine invertebrates are available in large numbers and high quality at Kahi Kai [14]. The embryo images in this database can serve as a benchmark for general image processing methods.

### Selection of representative genes

One gene was selected for the simulations from each gene cluster in Table 1 based on the number of available expression profiles for each gene. While the availability of a large number of profiles reduces the uncertainty in reconstructing the spatiotemporal expression patterns for that particular gene, it does not guarantee that this gene is representative of all other genes in the same cluster. To address this issue, it needs to be established, for each selected gene, whether this gene is an outlier in its own cluster.

### Effects of simplifications in the simulation model

The simulation model is formally fitted to 50 unique spatial points for 2 time points per gene (Figure 8B,C). However, all differential dynamics is observed between segments 25 and 45, while each gene reaches its final expression pattern from zero in roughly one time frame due to the intensity normalization. In this way, the useful information contained in the reference profiles is reduced to about 20 points for each of the 3 genes. The total information content is thus 60 points.

The original gene circuit formalism (Equation 1) includes n(n+5) optimization parameters for n fitted genes, which amounts to 24 optimization variables for our 3+1-gene simulation model. To limit the search space, i.e. the set of models that could potentially be evaluated during the optimization, the production rates and diffusion coefficients are excluded from the variation parameters. These modifications to the model reduce the number of variables to n(n+3), or 18 for the studied system. A lower number of variation parameters reduces the effects of overfitting, but also limits the solution space, i.e. the set of models that could be considered a good fit to the data.

The production rate in equation 1 determines the range of the production term. This means that a higher production rate allows a larger increase in gene product, and a higher production will generally result in higher product concentrations. In our simulations the reference input is normalized, so solutions with varying concentration maxima for the different genes do not contribute to a lower score. Therefore the variation of the production rates would not result in new regions of accessible solutions.

The diffusion coefficient determines the exchange rate of gene products between adjacent cells. A higher diffusion coefficient will smoothen the concentration profiles by decreasing large concentration differences between neighboring cells. In our best simulation the effect of removing diffusion is clearly visible for the spiked *FoxA* pattern at 25 hours (Figure 8D). Increasing the smoothness of the simulated patterns would result in a better fit.

A small, constant value for the diffusion coefficient that is the same for all genes can be a good alternative to completely neglecting diffusion. This will generate smooth profiles without expanding the search space, while inconsistencies with the real three-dimensional embryo morphology remain limited.

### Comparison to experiments

Tcf is an effector of the canonical Wnt pathway that forms a complex with β-catenin for its regulatory action. The effects of Tcf on the expression patterns of other genes in the blastula stage has been studied with knockdown experiments [18]. The genes *brachyury* and *foxA* are downregulated by NvTcf knockdown, while no significant effect is observed in the expression patterns of *snailA*, *snailB*, *sprouty*, *otxA*, *otxB* and *otxC*. Based on these knock-down experiments, this means that β-catenin/Tcf likely activates *brachyury* and *foxA*, but does not interact with the other genes. Our inferred GRN correctly includes activation of *foxA* and incorrectly predicts activation of *snail* by β-catenin.

The influence of Tcf on *twist* expression has not been addressed, because the knockdown study was limited to targets that are expressed in the blastula stage, and *twist* expression has not been observed before the late gastrula. The prediction from our regulation network that β-catenin/Tcf does not influence twist can be tested with a functional study.

### Comparison to another quantification approach

Our approach is similar to that of Crombach et al. [28] for *Drosophila*. The main features of their inferred gene networks are more reliable than ours, even though they include more genes and more parameters per gene in their optimizations. This is caused by different features in the source images, besides the obvious differences in image processing.

Their expression images are systematic time series, while our *in situ* hybridizations are obtained from several sources. Slight differences in staining procedures and microscopy settings can result in images with dissimilar properties.

Furthermore, the amount of images per gene in our sources is highly variable. This causes the clustering to be biased towards genes with many profiles. This bias could be diminished by averaging the patterns of each gene at identical time points before clustering, but for most images the exact development time is not available.

Both sources of uncertainty are diminished with the increased amount of hybridization images. A new series of hybridization images has recently become available from the Martindale lab in the Kahi Kai database. A repeated study including recent contributions would allow a more balanced clustering, a less biased gene selection and more accurate approximations of selected time points. Because the new measurements are systematic, it can even be sufficient to discard the nonsystematic sources.

### Robust results generated by the model

Some reliable results are obtained from the GRN model, despite its shortcomings. The cluster analysis of gene expression patterns confirms recent findings that genes in endomesoderm formation are mainly expressed in two regions. Moreover, many parameter sets in the gene circuit formalism are capable of simulating the major gene expression features, so the correct interactions that appear in the inferred GRN are probably necessary regulatory interactions to describe the main mechanism of *N. vectensis* gut patterning.

The main patterning mechanism includes maternal activation of genes in the oral pole (represented by foxA) and in the presumptive endoderm (represented by snail), and repression of the oral pole genes by the presumptive endoderm genes. β-catenin/Tcf is not necessary for snail expression, so snail requires another maternal activator. The interaction between snail and foxA has not been explored yet; a knockdown of snail is expected to upregulate foxA in the endodermal region.

### Suggested improvements to the model

The simulated genes have been selected based on a clustering of expression patters. However, this cluster analysis is unbalanced, with almost half of the profiles belonging to *snail* and *foxA*. A more homogeneous spread of observations over the genes selected for the correlation analysis should provide clusters that are populated more evenly. More systematic measurements would also provide more intermediate time points, allowing a more precise emergence of simulated profiles.

The proposed mechanism does not rely on the presence of twist; removing this gene from the simulation restricts the search space and may increase the sensitivity of the remaining parameters. Another strategy would be to increase the importance of a correctly simulated twist pattern to the overall fitness value. An increased weight for the simulated twist pattern can reduce the negative bias caused by the limited twist expression. Increasing the twist weight can be achieved by setting the twist similarity as a separate objective in a multi-objective optimization approach.

Replacing *twist* with a more suitable gene for simulation input will probably yield smoother simulated profiles and a better understanding of the core regulatory interactions than adding more genes to the current simulated system. Adding more genes will increase the number of parameters, and the simulated profiles can contain artifacts from irrelevant parameters.

### Gene expression quantification in other animals

With the spatio-temporal RNA data available for *N. vectensis*, a general procedure for improving GRNs can be designed. First, *in situs* from a gene expression database are quantified and a correlation analysis is performed. From this analysis, genes are chosen that represent major correlation clusters. For these genes, spatial distributions at fixed time points are constructed and prepared as input for network inference. Many optimization runs are performed and the resulting models are analyzed for their targeted properties, such as statistical relevance and parameter sensitivities. Based on this information, regulatory interactions among the simulated genes are proposed. These interactions, or their absence, are compared to experiments in literature or evaluated by additional experiments. This validation is then a new starting point to adjust the modeling framework or to change the set of simulated genes. A repeated series of parameter optimizations and model analyses should result in an improved gene interaction network.

The genes involved in the formation of various tissue types and organ systems have a high similarity across various organisms. Comparison of developmental regulation networks in different organisms can determine whether the regulatory interactions among these common genes are similar as well. These results may also test the hypothesis that an organism's outward complexity correlates with its number of regulatory interactions. In this light, it is interesting to note that previous observations have indicated that apparent complexity is independent from the organism's gene count [29].

Qualitative spatial expression maps have been drawn up for a wide variety of organisms in the blastula stage. If digital morphologies were constructed for these organisms, their spatial gene expression distribution could be quantified as well.

## Supporting Information

Dataset S1 **List of analyzed gene expression images.**
(ZIP)
