# Correction: A Computational Approach towards a Gene Regulatory Network for the Developing *Nematostella vectensis* Gut

**DOI:** 10.1371/journal.pone.0111161

**Published:** 2014-10-09

**Authors:** The PLOS ONE Staff

The first sentence of the second paragraph in the Spatial correlation and gene selection section in the Numerical analysis of the Methods appears incorrectly. “Parameter estimation” should appear as the second subheading of the Numerical analysis subsection.

Figure 1 is incorrect. The authors have provided a corrected version here.

**Figure 1:**
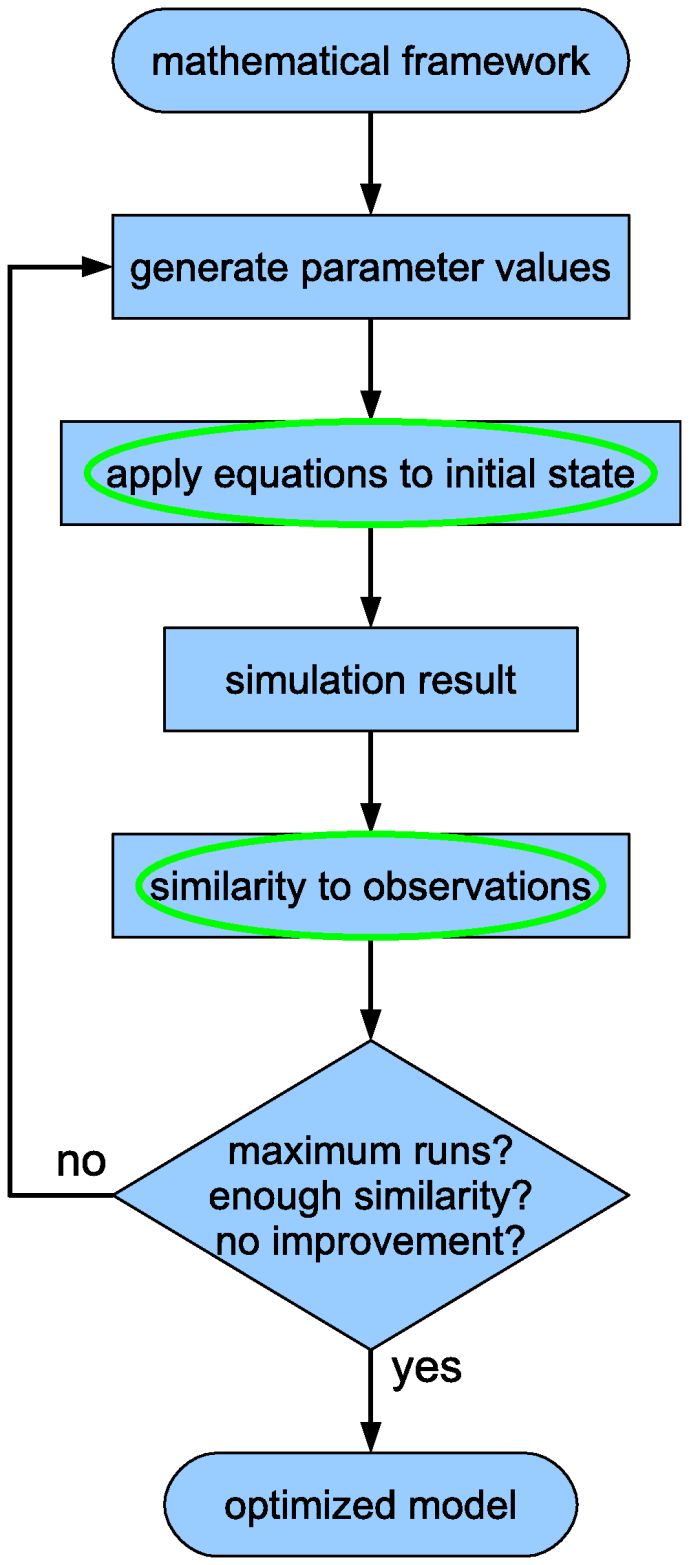
Overview of the modeling cycle. The modeling cycle starts with a framework of general mathematical equations. Initial parameter values are randomly generated or manually provided. These values are substituted into the general framework to define a specific set of equations. The equations are applied to the initial state of the system (usually derived from measurements) and produce intermediate and final states. These simulated states are compared to reference data and their similarity is determined. New parameter values are generated and new simulation runs are performed repeatedly, while stopping conditions are tested after each run (such as a maximum number of runs, a target similarity or a lack of improved similarity after multiple runs). As soon as a stopping condition applies, the cycle is terminated and the set of parameter values that results in the closest match with the observations is the optimized model. The steps that require quantitative data are encircled.
