# Supplementary material for: A Computational Approach towards a Gene Regulatory Network for the Developing Nematostella vectensis Gut
Source: PLoS One. 2014 Jul 30;9(7):e103341. doi: 10.1371/journal.pone.0103341 (PMC4116165; doi:10.1371/journal.pone.0103341)
Supplement: Dataset S1 — List of analyzed gene expression images. (ZIP) [file pone.0103341.s001.zip › finaldatasetS1.docx]

**Supporting Information**

**Dataset S1. List of analyzed gene expression images.** Left: spatial gene expression quantifications, right: gene expression images of *N. vectensis* embryos. Annotations in embryo images appear in original publications; the meaning of these annotations is irrelevant for quantification.


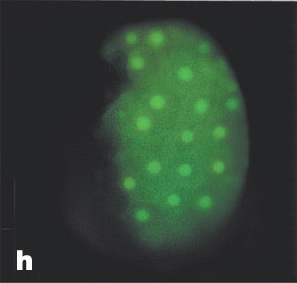


Bcat:b1 β-catenin in blastula - image 1 - oral right [32]


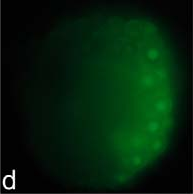


Bcat:b2 β-catenin in blastula - image 2 - oral right [33]


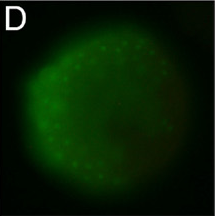


Bcat:b3 β-catenin in blastula - image 3 - oral left [27]


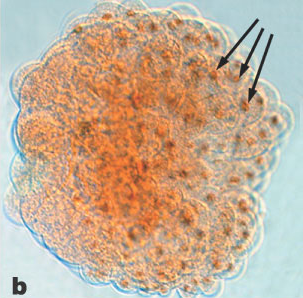


Bcat:bo β-catenin in blastula - orange staining - oral right [32]


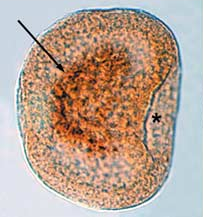


Bcat:27 β-catenin at 27 hpf - oral right [32]


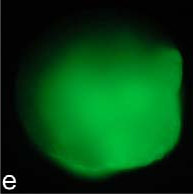


Bcat:50 β-catenin at 50 hpf - oral right [33]


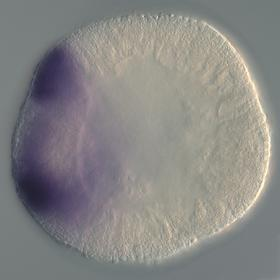


bra:bl brachyury in blastula - image 1 - oral left [14]


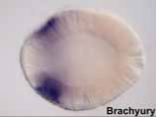


bra:bx brachyury in blastula - image 2 - oral left

(Martindale Lab, unpublished)


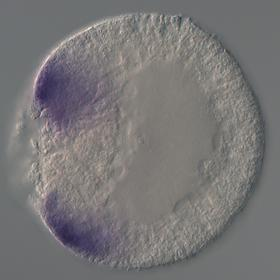


bra:24 brachyury at 24 hours - oral left [14]


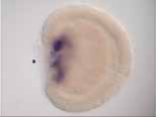


bra:27 brachyury at 27 hours - oral left

(Martindale Lab, unpublished)


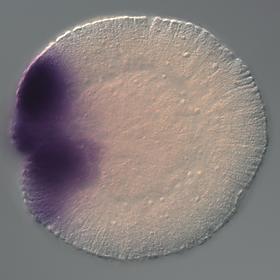


bra:30 brachyury at 30 hours - oral left [14]


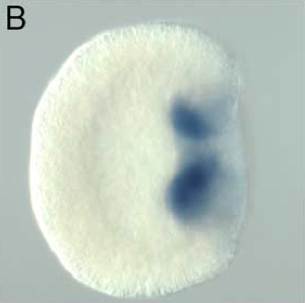


bra:35 brachyury at 35 hours - oral right [31]


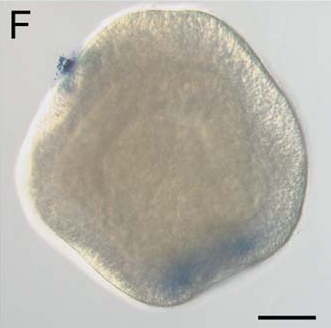


bra:50 brachyury at 50 hours - oral lower right [34]


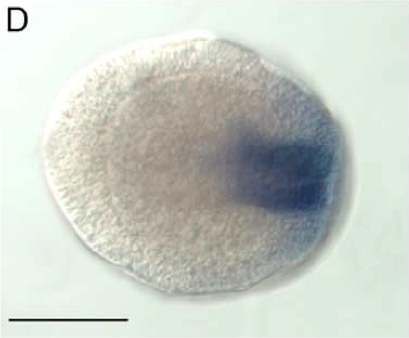


bra:70 brachyury at 70 hours - oral right [31]


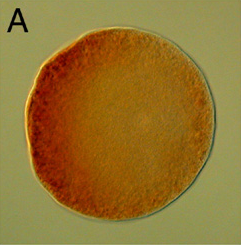


dsh:zy dishevelled in zygote - oral left [27]


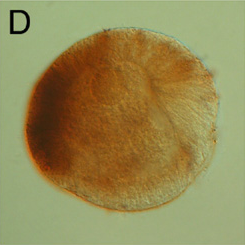


dsh:bl dishevelled in blastula - oral left [27]


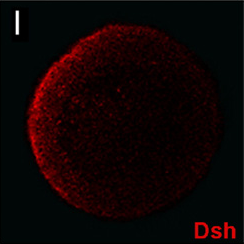


dsh:br dishevelled in blastula - red staining - oral left [27]


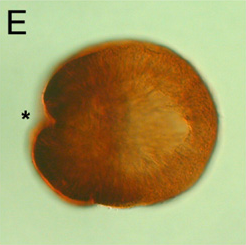


dsh:26 dishevelled at 26 hours - oral left [27]


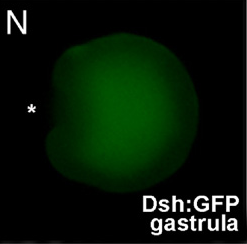


dsh:40 dishevelled at 40 hours - oral left [27]


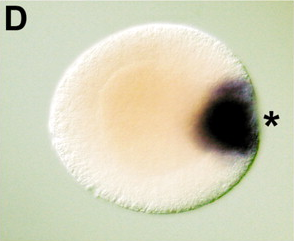


fork:22 foxA at 22 hours - oral right [17]


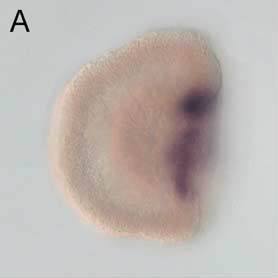


fork:26 foxA at 26 hours - oral right [31]


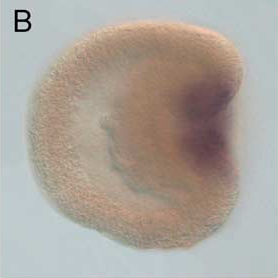


fork:27 foxA at 27 hours - oral right [31]


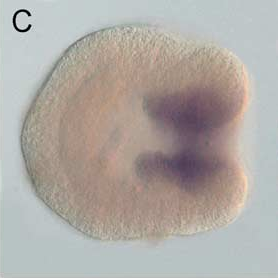


fork:30 foxA at 30 hours - oral right [31]


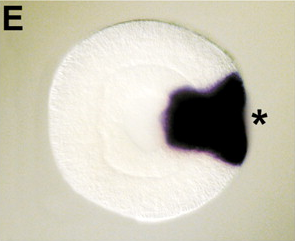


fork:32 foxA at 32 hours - oral right [17]


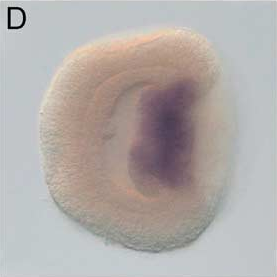


fork:33 foxA at 33 hours - oral right [31]


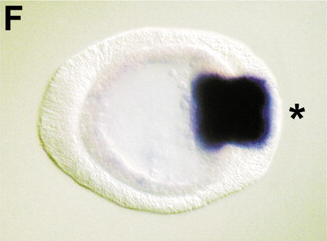


fork:36 foxA at 36 hours - oral right [17]


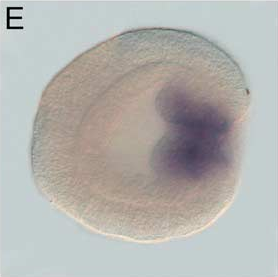


fork:40 foxA at 40 hours - oral right [31]


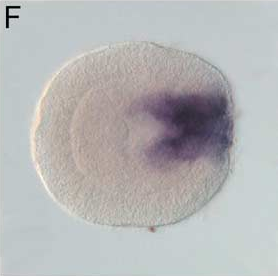


fork:42 foxA at 42 hours - oral right [31]


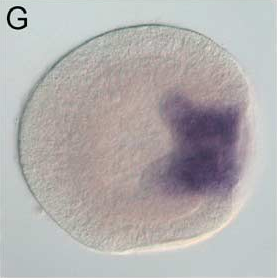


fork:44 foxA at 44 hours - oral right [31]


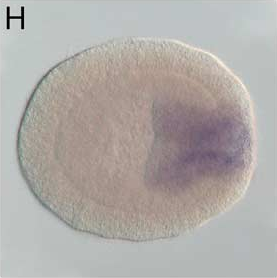


fork:54 foxA at 54 hours - oral right [31]


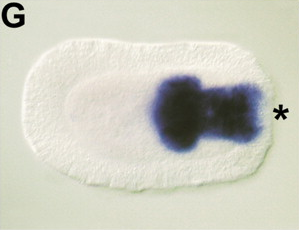


fork:60 foxA at 60 hours - oral right [17]


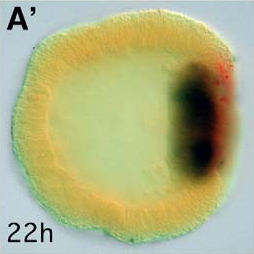


foxA:22 foxA at 22 hours - red staining - oral right [20]


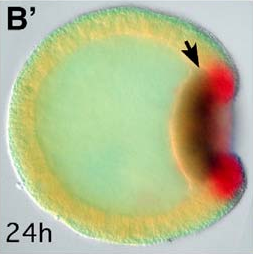


foxA:24 foxA at 24 hours - red staining - oral right [20]


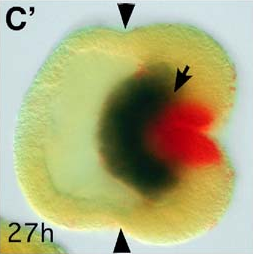


foxA:27 foxA at 27 hours - red staining - oral right [20]


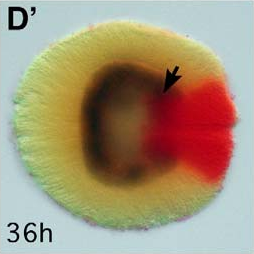


foxA:36 foxA at 36 hours - red staining - oral right [20]


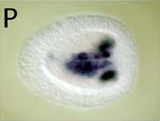


foxC:60 foxC at 60 hours - oral right [35]


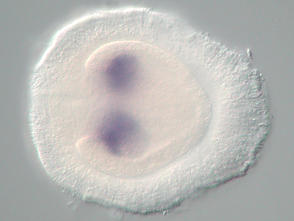


msx:50 msx at 50 hours - oral left [14]


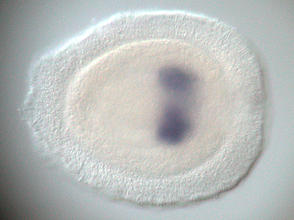


msx:60 msx at 50 hours - oral right [14]


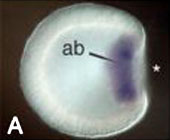


otxA:24 otxA at 24 hours - oral right [36]


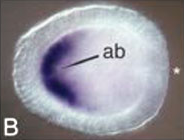


otxA:60 otxA at 60 hours - oral right [36]


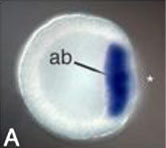


otxB:22 otxB at 22 hours - oral right [36]


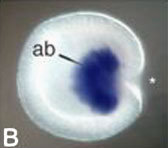


otxB:27 otxB at 27 hours - oral right [36]


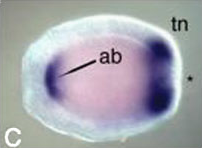


otxB:70 otxB at 70 hours - oral right [36]


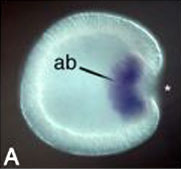


otxC:25 otxC at 25 hours - oral right [36]


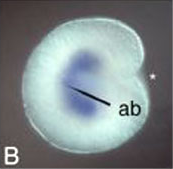


otxC:30 otxC at 30 hours - oral right [36]


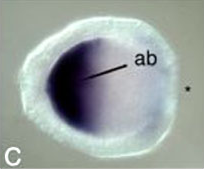


otxC:50 otxC at 50 hours - oral right [36]


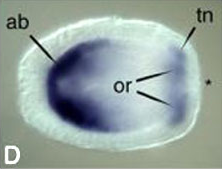


otxC:70 otxC at 70 hours - oral right [36]


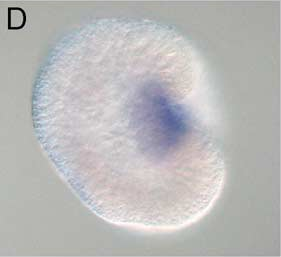


sna:eg snail in early gastrula - oral right [31]


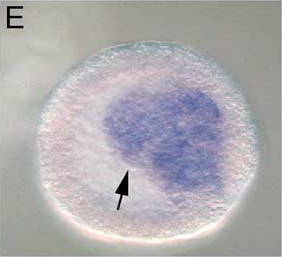


sna:mg snail in mid-gastrula - oral right [31]


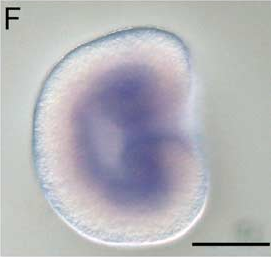


sna:lg snail in late gastrula - oral right [31]


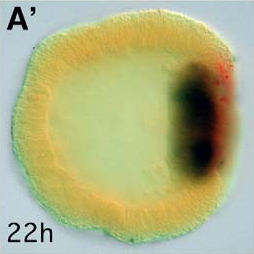


sna:22 snail at 22 hours - black staining - oral right [20]


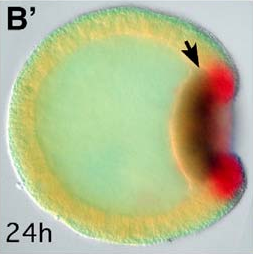


sna:24 snail at 24 hours - black staining - oral right [20]


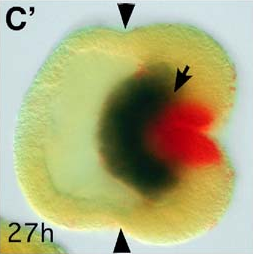


sna:27 snail at 27 hours - black staining - oral right [20]


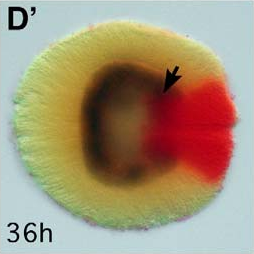


sna:36 snail at 36 hours - black staining - oral right [20]


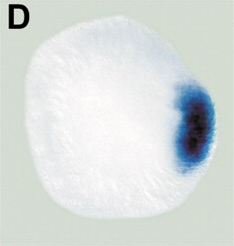


snail:bl snail in blastula - oral right [17]


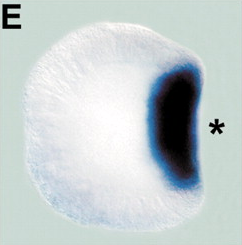


snail:23 snail at 23 hours - oral right [17]


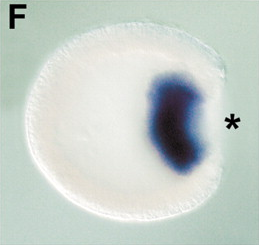


snail:24 snail at 24 hours - oral right [17]


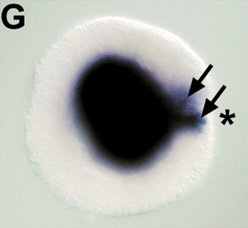


snail:30 snail at 30 hours - oral right [17]


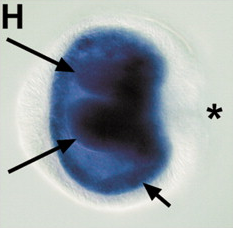


snail:45 snail at 45 hours - oral right [17]


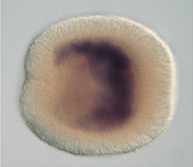


snail:48 snail at 48 hours - oral left [27]


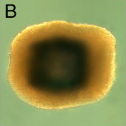


snail:50 snail at 50 hours - oral left [27]


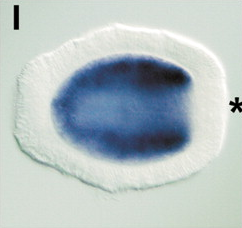


snail:60 snail at 60 hours - oral right [17]


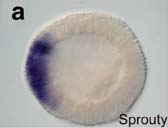


spr:bl sprouty in blastula - oral left [37]


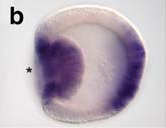


spr:25 sprouty at 25 hours - oral left [37]


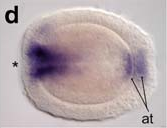


spr:50 sprouty at 50 hours - oral left [37]


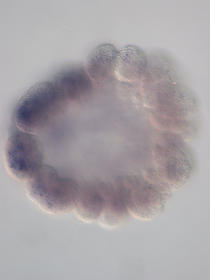


tcf:bl tcf in blastula - oral left [14]


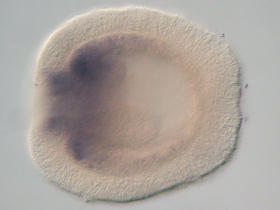


tcf:40 tcf at 40 hours - oral left [14]


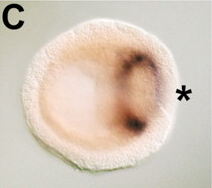


twi:38 twist at 38 hours - oral right [17]


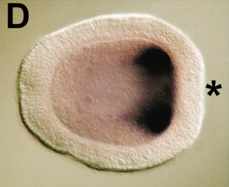


twi:54 twist at 54 hours - oral right [17]


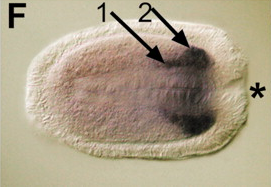


twi:70 twist at 70 hours - oral right [17]

**Additional references**

32. Wikramanayake AH, Hong M, Lee PN, Pang K, Byrum CA, et al. (2003) An ancient role for nuclear β-catenin in the evolution of axial polarity and germ layer segregation. Nature 426: 446-450.

33. Lee PN, Pang K, Matus DQ, Martindale MQ (2006) A WNT of things to come: evolution of Wnt signaling and polarity in cnidarians. Semin Cell Dev Biol 17: 157-167.

34. Scholz CB, Technau U (2003) The ancestral role of Brachyury: expression of NemBra1 in the basal cnidarian Nematostella vectensis (Anthozoa). Dev Genes Evol 212: 563-570.

35. Magie CR, Pang K, Martindale MQ (2005) Genomic inventory and expression of Sox and Fox genes in the cnidarian Nematostella vectensis. Dev Genes Evol 215: 618-630.

36. Mazza ME, Pang K, Martindale MQ, Finnerty JR (2007) Genomic organization, gene structure, and developmental expression of three clustered otx genes in the sea anemone Nematostella vectensis. J Exp Zool B Mol Dev Evol 308: 494-506.

37. Matus DQ, Thomsen GH, Martindale MQ (2007) FGF signaling in gastrulation and neural development in Nematostella vectensis, an anthozoan cnidarian. Dev Biol 313: 501-518.
